# Comparison of prognostic prediction models for rectal gastrointestinal stromal tumor

**DOI:** 10.18632/aging.103204

**Published:** 2020-06-20

**Authors:** Liu Jiaxin, Zhou Peiyun, Tang Zheng, Yuan Wei, Shen Shanshan, Ren Lei, Xing Zhengwen, Fang Yong, Gao Xiaodong, Xue Anwei, Shen Kuntang, Hou Yingyong

**Affiliations:** 1 Department of Pathology, Zhongshan Hospital, Fudan University, Shanghai, China; 2 Shanghai Chest Hospital, Shanghai Jiao Tong University, Department of Pathology, Shanghai, China; 3 Department of Liver Surgery, Liver Cancer Institute, Zhongshan Hospital, Fudan University, Key Laboratory of Carcinogenesis and Cancer Invasion of Ministry of Education, Shanghai, China; 4 Department of General Surgery, Zhongshan Hospital, Fudan University, Shanghai, China

**Keywords:** rectal GIST, nomogram, stratification

## Abstract

Background: Rectal gastrointestinal stromal tumors (RGISTs) are biologically characterized tumors that are relatively rare. Thus, few studies have reported a specific prognostic system for this subset of tumors but integrated it into parallel systems, such as small intestine. Our aim is to develop a new predictive staging system nomogram (named FD-ZS system) for RGISTs.

Results: Tumor size and mitotic rate were independent risk factors for tumor recurrence in RGISTs according to univariate and multivariate survival analyses. A prognostic predictive nomogram was developed, and a cut-off value of 65 points was calculated by X-tile to discriminate risk based on tumor size and mitotic rate. The C-indices for the FD-ZS, FD-Hou, NIH, and WHO systems were 0.706, 0.693, 0.687, and 0.680, respectively.

Conclusion: In the present study, a concise two-tier grading system (FD-ZS) for prognostic prediction of RGISTs that is simpler to several reported systems was developed, and a cut-off value was established to help RGIST patients determine whether to undergo adjuvant imatinib treatment.

Methods: A nomogram was employed, and its predictive accuracy and discriminative ability were determined by concordance index (C-index) and calibration curve analyses. The nomogram was then compared with three stratification systems used for GISTs (FD-Hou, NIH, and WHO).

## INTRODUCTION

Gastrointestinal stromal tumors (GISTs) are the most common mesenchymal tumors of the gastrointestinal tract and represent approximately 1~3% of all primary gastrointestinal malignancies [1, 2]. Their clinical and pathological features differ significantly in terms of sites (stomach, small intestine, colon and rectum) [3, 4]. Rectal GISTs (RGISTs) account for less than 5% of all GIST cases and are associated with a poor survival prognosis [5–6].

Most GISTs contain a mutation in the KIT proto-oncogene, or less frequently in the PDGFRA gene, and are usually responsive to the tyrosine kinase inhibitor imatinib mesylate (IM) (Gleevec, Novartis Pharmaceuticals Corporation, Basel, Switzerland) [7, 8]. With the advent of molecular-targeted therapy, the treatment of metastatic or unresectable GISTs has generated excellent results. Perioperative IM therapy may be particularly beneficial for RGISTs, and some have reported that neoadjuvant chemotherapy can reduce tumor volume, thereby facilitating surgical resection or increasing the likelihood of organ retention and further improving patient prognosis [9–11]. Based on these data and the presence of KIT exon 11 mutations in most RGISTs (KIT exon 11 is more sensitive to IM than other mutations, such as KIT exon 9 or PDGFRA), preoperative IM therapy is recommended for RGISTs in the majority of advanced cases. The surgical approach for RGIST removal has also changed significantly from radical excision to local resection or low anterior resection [12–15].

When screening patients who may benefit from IM-assisted therapy, the most important factor is estimation of the risk of recurrence after primary GIST resection. Risk stratification of GISTs is mainly based on tumor size, mitotic activity and location, the most well-recognized indicators of recurrent or metastatic risk [16]. The American College of Surgeons Oncology Group (ACOSOG) Z9001 trial also confirmed these three prognostic indicators [17]. However, due to a paucity of research and insufficient studies, no individual or optimal staging system exists for RGISTs. Two commonly used staging systems for prognosis are the modified National Institutes of Health consensus criteria (NIH criteria of 2008) and the WHO Classification of Tumors of the Digestive System consensus criteria (WHO criteria of 2013) (Table 1) [18, 19]. Neither of these classifications provides a quantifiable risk of recurrence for individual patients, which limits the ability of stratifying the risk ratio of RGISTs compared to other GISTs. A nomogram developed at Memorial Sloan-Kettering Cancer Center (MSKCC) has been employed to predict the risk of tumor recurrence after gross surgical resection of GIST [20]. A moderate risk of RGISTs was revealed for the stomach and small intestine, yet only 14 colonic or rectal GISTs were included in the nomogram, potentially reducing the productive and predictive value for RGISTs. Therefore, it is necessary to develop a specific stratification system for RGIST recurrence to identify patients with a high risk of recurrence who can receive individualized treatment according to their specific circumstances.

**Table 1:** Staging systems for assessing risk of RGIST and cases entering.

FD-ZS	non-malignant	I		≤65 nomogram points	38
	malignant	II		>65 nomogram points	32
FD-Hou	non-malignant	I	0	*liver metastassis	29
	malignant	II	≥1	*peritoneal dissemination	41
				*lymph node metastasis	
				*vascular infiltration	
				*fatty infiltration	
				*nerve infiltration	
				*mucosal infiltration	
				*mitoses≥10/50HPF	
				*muscle infiltration	
				*coagulative necrosis	
				*perivascular pattern	
				*severe nuclear atypia	
WHO 2013	Benign(1;2;3a)	I		≤2 cm and ≤5 mitotic index;	34
				>2 cm and ≤10 cm, and ≤5 mitotic index	
	Intermediate(4)	II		≤2 cm and >5 mitotic index	3
	Malignant(3b;5;6a;6b)	III		>10 cm and ≤5 mitotic index;	33
			>2 cm and ≤5 cm, and >5 mitotic index;	
				>5 cm and >5 mitotic index	
NIH 2008	Very low	I		≤2 cm and ≤5 mitotic index	20
	Low	II		>2 cm and ≤10 cm, and ≤5 mitotic index;	12
				≤2 cm and >5 mitotic index	
	Intermediate	III		>10 cm and ≤5 mitotic index;	4
				>2 cm and ≤5 cm, and >5 mitotic index	
	High	IV		>5 cm and >5 mitotic index	34

In this study, we developed a new, effective statistical model for individual RGIST patients based on a moderately large cohort. Our aim was to predict the risk of tumor recurrence after gross surgical resection of localized primary RGISTs combined with or without postoperative chemotherapy. Furthermore, we compared this model with the NIH, WHO and FD-Hou (a staging system developed at Fudan University by Hou Yingyong based on 12 clinicopathological parameters, Table 1) staging systems [21, 22], and we confirmed the role of IM in the management of RGISTs.

## RESULTS

### Demographics and clinical characteristics of the 105 eligible patients

Of these patients, 45 (42.85%) received tyrosine kinase inhibitor treatment, and 60 (57.15%) underwent surgery with no pharmacological treatment before the first relapse. We describe the patient demographics and clinical characteristics of the four groups separately in Table 2. Based on immunochemistry, more than 90% of the tumors exhibited DOG-1, CD117, and CD34 positivity. Of the 105 patients, 89 (84.76%) were genetically tested and found to have KIT exon 11 mutations (66, 74.16%), KIT exon 9 mutations (13, 14.61%), or a wildtype genotype (10, 11.23%). No patients exhibited PDGFRA mutations.

**Table 2:** Demographics and clinical characteristics of the 105 eligible patients.

**Characteristic**	**Variable**	**Resection(n=60)**	**Adjuvant(n=10)**	**Imatinib(n=10)**	**Neoadjuvant(n=25)**
Sex	Female	19(31.67%)	6(60.00%)	3(30.00%)	8(32.00%)
	Male	41(68.33%)	4(40.00%)	7(70.00%)	17(68.00%)
Age	years±SD	57.38±13.10	50.90±13.76	58.30±14.17	50.20±11.58
Center	Out-zhongshan	17(28.33%)	9(90.00%)	3(30.00%)	12(48.00%)
	Zhongshan	43(71.67%)	1(10.00%)	7(70.00%)	13(52.00%)
Mutation	Wild type	10(21.74%)	0(0.00%)	0(0.00%)	0(0.00%)
	KIT exon 9	5(10.87%)	2(20.00%)	2(22.22%)	4(16.67%)
	KIT exon 11	31(67.39%)	8(80.00%)	7(77.78%)	20(83.33%)
IM after the first surgery	No	60(100.00%)	0(0.00%)	0(0.00%)	6(24.00%)
	Yes	0(0.00%)	10(100.00%)	0(0.00%)	19(76.00%)
	Other^b^	0(0.00%)	0(0.00%)	10(100.00%)	0(0.00%)
Time of IM after the first surgery	months±SD	0.00±0.00	26.00±19.24	0.00±0.00	18.74±12.82
Tumor size at diagnosis	cm±SD	3.74±3.40	4.08±1.26	7.91±3.25	6.13±2.02
Current tumor size^c^	cm±SD			5.56(2.81)	4.50(1.37)
Procedure of treatment	LE	36(60.00%)	6(60.00%)		
	APR/TPE	17(28.33%)	1(10.00%)		
	Other^a^	7(11.67%)	3(30.00%)		
Follow-up(months)	Median	80	70	32	57
	Range	11-235	12-122	15-60	12-167

Resection: resection only including 10 cases had taken IM after the first relapse; Adjuvant: resection firstly combined with adjuvant therapy

Imatinb: IM survival with tumor after diagnosis; Neoadjuvant: preoperative IM therapy

^a^For Kraske or surgical procedure unknown

^b^For some patients(n=10) with imatinib not surgery

^c^For patients with imatinib, size was based on imaging upon surgery or follow-up who did not receive surgery

LE: local excision, APR: abdominoperineal resection, TPE: total pelvic exenteration

### Imatinib has an effect on OS based on 105 patients

**Figure 1:**
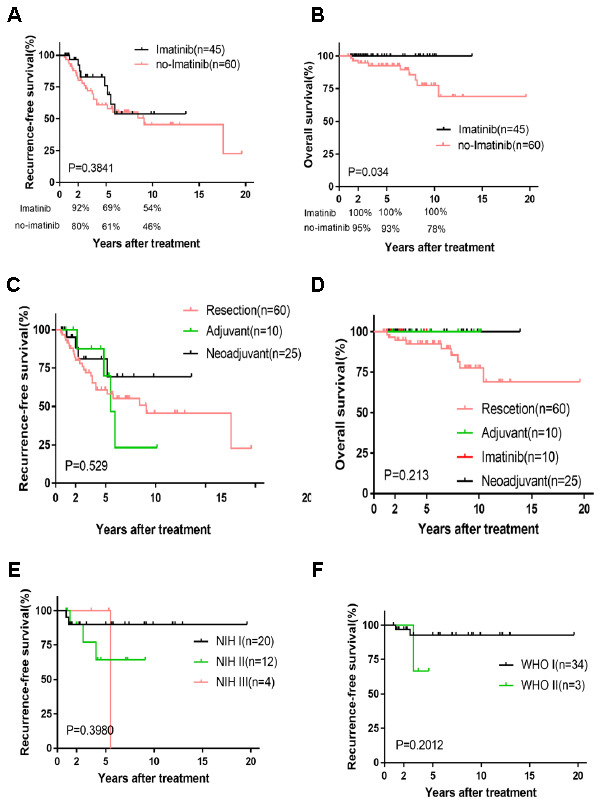
**Kaplan-Meier survival plot of RFS and OS based on imatinib and four treatments and the survival curve of RFS based on relatively lower risk.** RFS and OS of imatinib and no-imatinib (**A**, **B**); RFS and OS of four treatments (**C**, **D**); RFS of NIH I, II, III and WHO I, II (**E**, **F**).

The Kaplan-Meier curve of overall survival (OS) showed that IM treatment (45 cases) had a significant benefit compared with no-IM treatment (60 cases) (*P*=0.034), whereas no difference was found for recurrence-free survival (RFS) (*P*=0.3841). Of note, the 2-, 5-, and 10-year RFS rates of the IM group (92%, 69%, and 54%) were higher than those of the no-IM group (80%, 61%, and 46%), which were similar to the OS rate results (IM group [100%, 100%, and 100%], no-IM group [95%, 93%, and 78%]) (Figure 1A and 1B). Our findings suggest that IM is not a statistically independent prognostic factor of RFS. Furthermore, we analyzed 3 subgroups based on the treatment regimens of resection only and neoadjuvant therapy, adjuvant and neoadjuvant therapy, and resection only and adjuvant therapy. Consistent with previous reports, tumor size and mitosis were the dominant risk factors [16] (Supplementary Tables 1–4). The Kaplan-Meier survival plot indicated insignificant differences in OS (*P*=0.529) and RFS (*P*=0.213) for the four treatments (Figure 1C and 1D).

### Nomogram and validation based on 70 selected patients

**Figure 2:**
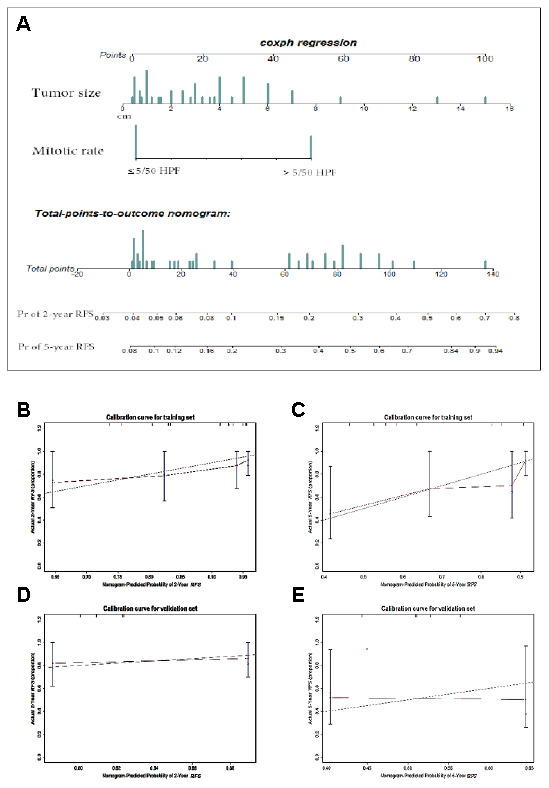
**Nomogram and validation to predict the probabilities of 2-year and 5-year recurrence-free survival.** To use the nomogram (**A**), an individual patient’s value is located on each variable axis, and a line is drawn upward to determine the number of points received for each variable value. The sum of these points is located on the Total points axis, and a line is drawn downward to the survival axes to determine the likelihood of 2- or 5-year RFS. Tumor size(cm), mitotic rate (≤5 or >5 mitoses per 50 HPFs). The calibration curve for predicting patient survival at (**B**) 2 years and (**C**) 5 years in the training set and at (**D**) 2 years and (**E**) 5 years in the validation set. Nomogram-predicted probability of RFS is plotted on the x-axis; actual RFS is plotted on the y-axis.

To develop a predictive nomogram for RGISTs, 70 cases without any treatments before surgery were selected and 44 treated at FDZS were applied in the training cohort, thus 26 from the consultation files as the validation cohort. We examined the following parameters by Cox regression analyses: age, sex, treatment procedure, mutation, IM after the first surgery, time of IM after the first surgery, tumor size at diagnosis, and mitotic rate. Univariate and multivariate analyses demonstrated tumor size (assessed as a continuous variable) and mitotic rate (with a breakpoint of ≤5 or >5 mitoses per 50 HPFs) to be independent risk factors for tumor recurrence and OS (Supplementary Table 5). The prognostic nomogram integrated the independent factors to predict RFS among RGIST patients (Figure 2A), and nomogram-assigned points were used to predict the 2-year and 5-year RFS probabilities for each patient. The concordance probability of the nomogram was 0.706, indicating that the nomogram predicted the correct outcome 70.6% of the time for randomly selected patients (Table 3). The calibration plot for the probability of survival at 2 or 5 years after treatment showed optimal agreement between the nomogram prediction and the actual observation (Figure 2B and 2C). The cut-off value of 65 points was calculated by X-tile, which parsed the risk into a nonmalignant group (≤65 points) and a malignant group (>65 points). Thus, a complete and concise, two-tier staging system was developed, which was named the FD-ZS staging system.

**Table 3:** Concordance probabilities of the nomogram compared with other commonly used staging systems.

	**Nomogram Concordance**	**FD-Hou Concordance**	**WHO Concordance**	**NIH Concordance**
RFS	0.706	0.693	0.680	0.687
OS	0.872	0.762	0.810	0.799

Nomogram:FD-ZS

In the validation cohort, the concordance index (C-index) of the nomogram for predicting RFS was 0.557, and a calibration curve showed good agreement between the predicted and observed probability of 2-year and 5-year RFS (Figure 2D and 2E). The C-index of the nomogram for OS was 0.872, which indicated optimal agreement between the nomogram prediction and the actual observation (Supplementary Figure 1). However, validation of the nomogram was not observed, which may be attributable to the low mortality rate at the end of follow-up.

### Comparison of predictive accuracy for RFS between the novel nomogram and conventional staging systems

**Figure 3:**
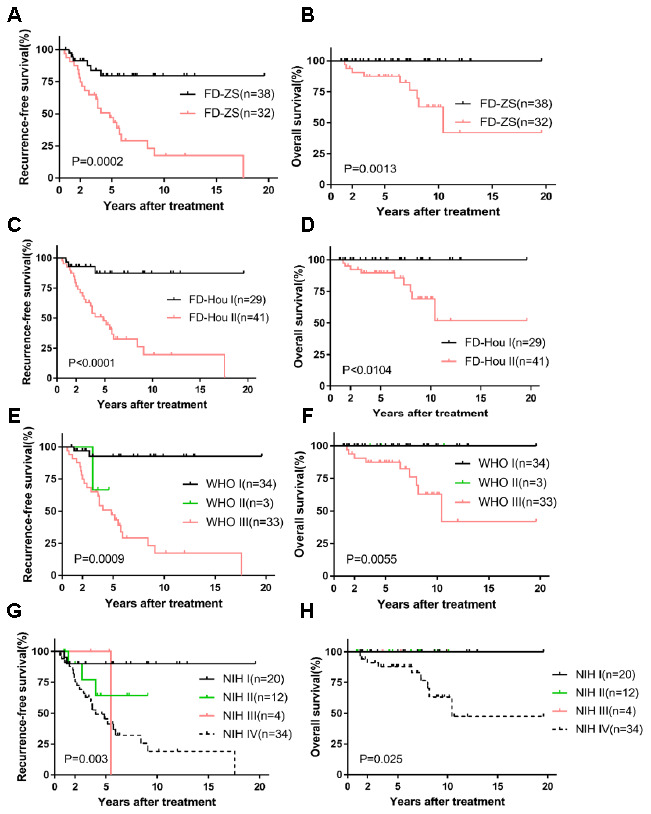
**Kaplan-Meier survival plot of RFS and OS based on FD-ZS, FD-Hou, WHO and NIH staging system.** RFS and OS of FD-ZS (**A**, **B**); RFS and OS of FD-Hou (**C**, **D**); RFS and OS of WHO (**E**, **F**); RFS and OS of NIH (**G**, **H**).

Our nomogram displayed better accuracy in predicting both short- and long-term RFS in the training cohort (0.706) than did the WHO staging system (0.680) and the NIH criteria (0.687). The accuracy of the FD-Hou staging system (0.693) fell between those of the novel nomogram and the other two systems. For OS, the nomogram predictive value (0.872) was highest compared to the FD-Hou (0.762), WHO (0.810), and NIH (0.799) systems (Table 3). The Kaplan-Meier curve of RFS showed good prognostic stratification for the four staging systems (FD-ZS [*P*=0.0002], FD-Hou [*P*<0.0001], WHO [*P*=0.0009] and NIH [*P*=0.003]), and that of OS also displayed significant stratification (FD-ZS [*P*=0.0013], FD-Hou [*P*<0.0104], WHO [*P*=0.0055] and NIH [*P*=0.025]) (Figure 3). The NIH and WHO staging systems showed good prognostic stratification for patients with relatively low NIH-based risk grades (very low risk, low risk, and intermediate risk, 36 cases) and high-risk grades (34 cases) and WHO-based nonmalignant grades (benign, intermediate, 37 cases) and malignant grades (33 cases). However, both the NIH and WHO criteria were unsatisfactory in stratifying RGIST patients among very low risk, low risk, and intermediate risk (*P*=0.3980), as well as between benign and intermediate (*P*=0.2012) classifications, suggesting that it is unnecessary to focus on subgroup stratification for lower-risk RGIST cases and that high-risk patients should mainly be screened (Figure 1E and 1F).

The Kaplan-Meier curves for OS and RFS were used to analyze malignant groups from the FD-ZS (II), FD-Hou (II) and WHO (III) systems and the highly malignant group of the NIH (IV) system with regard to patients with and without postoperative IM treatment (Supplementary Figure 2). No statistically significant difference was found in RFS for the four risk stratification methods. However, the Kaplan-Meier curve of OS demonstrated a possible trend for the FD-ZS staging system (*P*=0.0841) and WHO criteria (*P*=0.0841), indicating that postoperative chemotherapy can improve prognosis. The NIH criteria (*P*=0.1661) and the FD-Hou system (*P*=0.1346) exhibited a slightly weaker prognostic trend.

## DISCUSSION

In the past twenty years, GISTs have been the most successfully treated type of solid tumor by molecular-targeted therapy. Molecular targeting of the KIT gene and the PDGFRA gene can effectively control recurrent and metastatic high-risk GIST and has become a model for molecular-targeted therapy of solid tumors. The response of GISTs to IM significantly depends on the mutation sites in KIT and PDGFRA. KIT exon 11 mutations may be markedly responsive to IM, whereas KIT exon 9 mutations may require a higher dose of IM. Additionally, wildtype tumors and PDGFRA-mutated tumors show ineffective responses to IM [23, 24]. Therefore, GISTs have become a model for individualized treatment of tumors. Neoadjuvant therapy and adjuvant therapy might benefit to RGIST patients and extend their RFS and OS [9, 11, 25]. As the incidence of RGIST is exceedingly low, very scant information is available on RGISTs. Due to the use of IM and the specificity of rectal anatomy (locally progressive RGISTs may be bulky due to the pelvic space and often adhere to the pelvic floor), the majority of patients currently choose to undergo molecular-targeted therapy instead of direct surgery after diagnosis of an RGIST. Unless the tumor is small, surgery is expected to preserve the sphincter. Therefore, the natural history of RGISTs has been less extensively investigated. This study has an apparent strength due to the relatively large number of primary specimens.

The aforementioned studies have confirmed the role of IM in RGISTs. In our study, the Kaplan-Meier curve for RFS indicated no significance between the IM and no-IM groups. Of note, we found a statistically significant difference in OS based on IM; none of the patients in the IM group incurred an incident event as of the last follow-up. The survival curve of RFS indicated that taking IM may be better than not taking IM.

Our department of pathology has accumulated numerous GIST samples because of our long-term attention to the clinical treatment of GISTs [21, 22, 26]. Based on the collected samples, our study used newer statistical tools to focus on the treatment and prognosis of RGISTs. Previous reports have indicated the specific biological and prognostic features of RGISTs compared to GISTs at other locations, as well as their high local recurrence rate [5, 6]. Moreover, in two commonly used staging systems (NIH and WHO), an appropriate grading system for RGISTs was not developed, but rather RGISTs were incorporated into the system used for small intestinal GISTs. Our research led to the development of a nomogram model to predict the individual prognosis of RGIST patients. Tumor size was consistent with that in previous reports and was easy to interpret, and the mitotic rate was assessed once again by two experienced pathologists. The concordance probability of the nomogram showed better performance than that of the NIH, WHO and FD-Hou systems. The cut-off value of 65 points was selected to stratify risk into low and high. An obvious stratification in the four staging systems was observed by Kaplan-Meier curves, but we did not find a significant difference among the groups with relatively lower risk RGISTs using the WHO or NIH systems. Because of the low incidence of RGISTs, the use of multi-grade stratification systems, and the low number of cases within each grade, individual grades may not have included any cases; this may be especially true for the WHO system, which has 8 grades of GIST. Moreover, classification of the malignant group in the WHO system (3b, 5, 6a, 6b) was not superior to that of the nomogram, which alerted us to the necessity of concise estimation of RGIST classification. Our study also suggested that it is unnecessary to focus on subset stratification for relatively lower-risk RGIST cases and that mainly high-risk patients should be screened. The FD-Hou staging system uses a variety of morphological indicators and thus requires a GIST pathologist with specific training. High-risk malignances in the four stratifications can all benefit from treatment with the tyrosine kinase inhibitor IM. The prognostic nomogram provides a better predictive performance for individual patients than the prediction resulting from dividing patients into large groups. Nomograms in other studies quantified the risk of tumor recurrence after complete resection as a continuous variable, and we used a cut-off value as a threshold for tumor recurrence to help select patients for IM treatment [20, 27, 28].

No difference in RFS was observed for high-risk patients, regardless of whether they received IM treatment after the first surgery. In the analysis of OS, high-risk patients who received IM after the first surgery showed a better prognosis, though statistical significance was not reached. This result may be due to the low number of patients with IM therapy after the first resection in the cases we included because our samples were from both the IM era and the pre-IM era, in which IM was unavailable. Ultimately, after applying the nomogram, the survival curve of the IM subgroup in the malignant group exhibited a possible trend for improved prognosis. Therefore, we recommend that the malignant group (>65 points) should actively receive IM therapy and that the nonmalignant group (≤65 points) may require long-term follow-up.

As our staging system is based on two indicators of tumor size and mitotic rate, it is only suitable for patients who have not received any treatment before surgery. If the patient has been treated before surgery, the tumor size and the mitotic count of postoperative pathological specimens may be affected. Hence, this model can be used to predict the patient's recurrence risk after surgery in GIST cases originating in the rectum and without any treatment before surgery, as long as the clinician records the tumor size of the patient during surgery and the mitotic count (50 HPFs) of the postoperative pathological specimen.

There are several limitations in this study. First, the validation cohort C-indices were only 0.557 (RFS) and 0.600 (OS), which may be attributed to the small sample size (n=26). Second, the two factors used to develop the nomogram might not have been measured uniformly across institutions. Tumor size may be affected by fixation or imaging differences, and the mitotic rate can be largely affected by subjective factors; therefore, experienced pathologists are still needed to assess the validity of this novel nomogram. Third, our study only recruited patients in China, and the results may not be applicable to other countries, especially Western countries. Finally, with a retrospective study, unknown or unobserved sources may result in information bias.

## CONCLUSION

Our study pioneers the development of a concise two-tier grading system (FD-ZS system) for prognostic prediction of RGISTs based on tumor size, mitotic rate and a cut-off value to help RGIST patients determine whether to undergo adjuvant IM treatment. The predictive ability of the system is simpler to that of current international systems, including the NIH system (2008), the WHO system (2013), and the FD-Hou clinicopathological parameters staging system. The FD-ZS staging system may help to more concisely guide the combined use of surgery and molecular-targeted therapy in RGIST and improve the prognosis of RGIST patients. In addition, we found that IM affected the OS of RGIST patients and may result in longer survival.

## MATERIALS AND METHODS

### Participants and criteria

**Figure 4:**
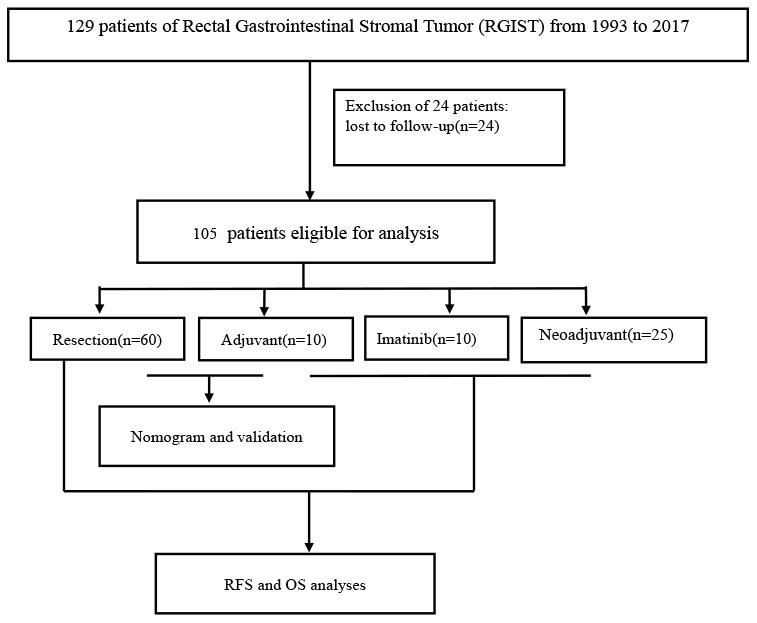
**Study flow chart.**

The study enrolled 129 patients who were histologically diagnosed with GISTs originating in the rectum via needle biopsy or surgery at Zhongshan Hospital, Fudan University (FDZS) between 1993 and 2017. All specimens were confirmed by two experienced pathologists. The mitotic count data represent the total number of mitotically active cells from 50 consecutive high-power microscopic fields (HPFs), and tumor size was based on imaging or formalin fixation upon surgery. Twenty-four patients were lost to follow-up and excluded. The 105 patients eligible for analysis included those who underwent resection only (60, 57.14%, patients who did not receive any treatment after the first surgery until the tumor recurred and did not receive any treatment before the surgery, cohort 1), resection combined with adjuvant therapy (10, 9.52%, patients who did not receive any treatment before surgery, and received IM after the first surgery until tumor recurrence, cohort 2), IM survival with tumor (10, 9.52%, patients who received IM treatment after diagnosis by needle biopsy, but no surgical treatment as of follow-up, 2 of them had IM intolerance, cohort 3), and neoadjuvant IM treatment (25, 23.81%, patients who received IM treatment before surgery, 2 of them had IM intolerance and changed to sunitinib, cohort 4). Forty-one patients treated at other hospitals were collected through the clinical consultation files of Zhongshan Hospital between 2004 and 2017. Sixty-four patients from FDZS were hospitalized between 1993 and 2017. The nomogram was developed based on 44 patients treated at FDZS between 1993 and 2017, and the validation cohort (26 cases) consisted of cases from the consultation files. All data regarding the patients’ demographics, morbidity, postoperative mortality, and histological results were obtained from the hospital medical system or consultation files. The follow-up information for patients was provided by the referring pathologists and clinicians or obtained from the patients and their family members through direct contact via mailing and telephone. The median follow-up time was 59.75 months (range 11-235 months), and the final follow-up date was June 2018. The primary research endpoint was the death of the patient or the end of follow-up; the secondary endpoint was follow-up dropout. OS was defined as the period from diagnosis until death due to any cause. RFS was defined as the duration from surgery until the date of RGIST recurrence. The study was conducted and simplified according to the flow chart shown in Figure 4.

### Statistics

Cox proportional hazards regression models were constructed to select prognostic factors, and R packages (rms, Hmisc, lattice, survival, Formula, ggplot2, regplot) were loaded into R version 3.5.1 to plot the nomogram [29]; concordance probability was employed to estimate the superiority of the model. The method of asymptotic significance level calibration was tested with 1000 bootstrap resamples. Calibration was assessed by plotting the predicted probabilities against the actual outcomes [30]. X-tile was used to select the cut-off point [31]. GraphPad Prism 6 software was applied to draw the Kaplan-Meier curves of RFS and OS.

### Ethics approval

The study with clinical data was approved by the Ethics Committee of the Zhongshan Hospital, Fudan University.

## Supplementary Material

Supplementary Figures

Supplementary Table 1

Supplementary Table 2

Supplementary Table 3

Supplementary Table 4

Supplementary Table 5
