# Supplementary material for: Comparison of prognostic prediction models for rectal gastrointestinal stromal tumor
Source: Aging (Albany NY). 2020 Jun 20;12(12):11416–30. doi: 10.18632/aging.103204 (PMC7343501; doi:10.18632/aging.103204)
Supplement: Supplementary Figures [file aging-12-103204-s006..pdf]

## SUPPLEMENTARY FIGURES

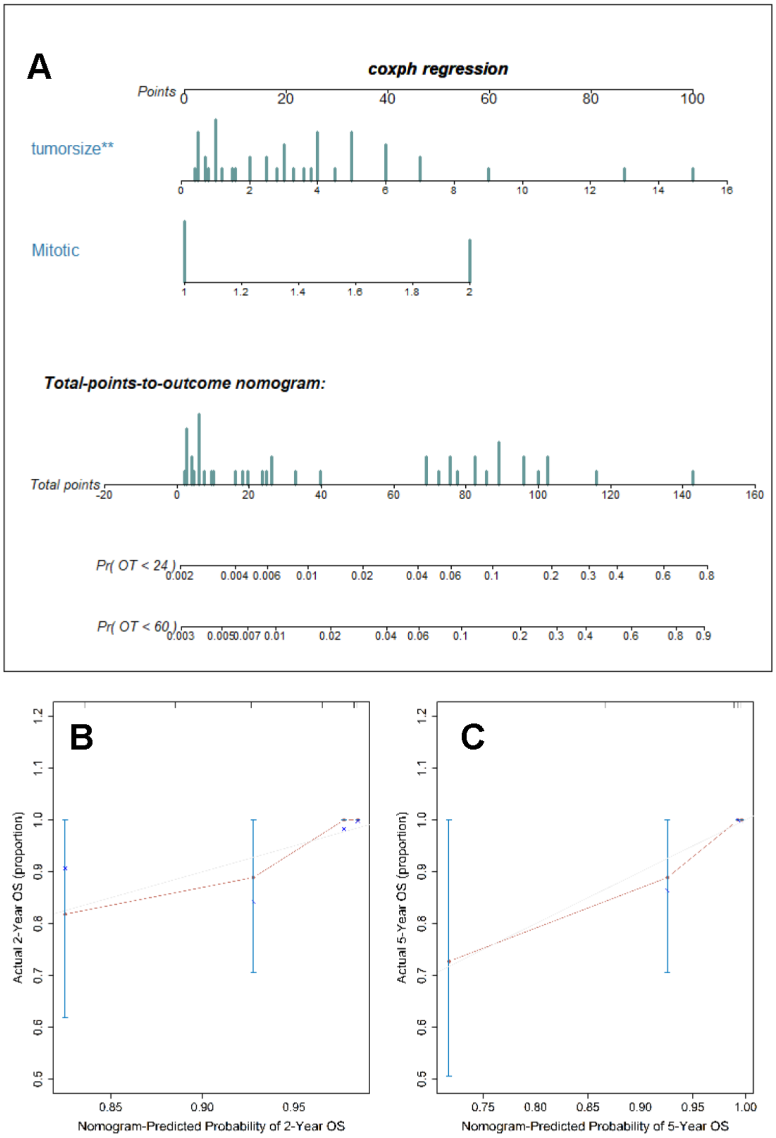


**Supplementary Figure 1. Nomogram and validation to predict the probabilities of 2-year and 5-year overall survival** (**A**). The calibration curve for predicting patient survival at (**B**) 2 years and (**C**) 5 years in the training set.

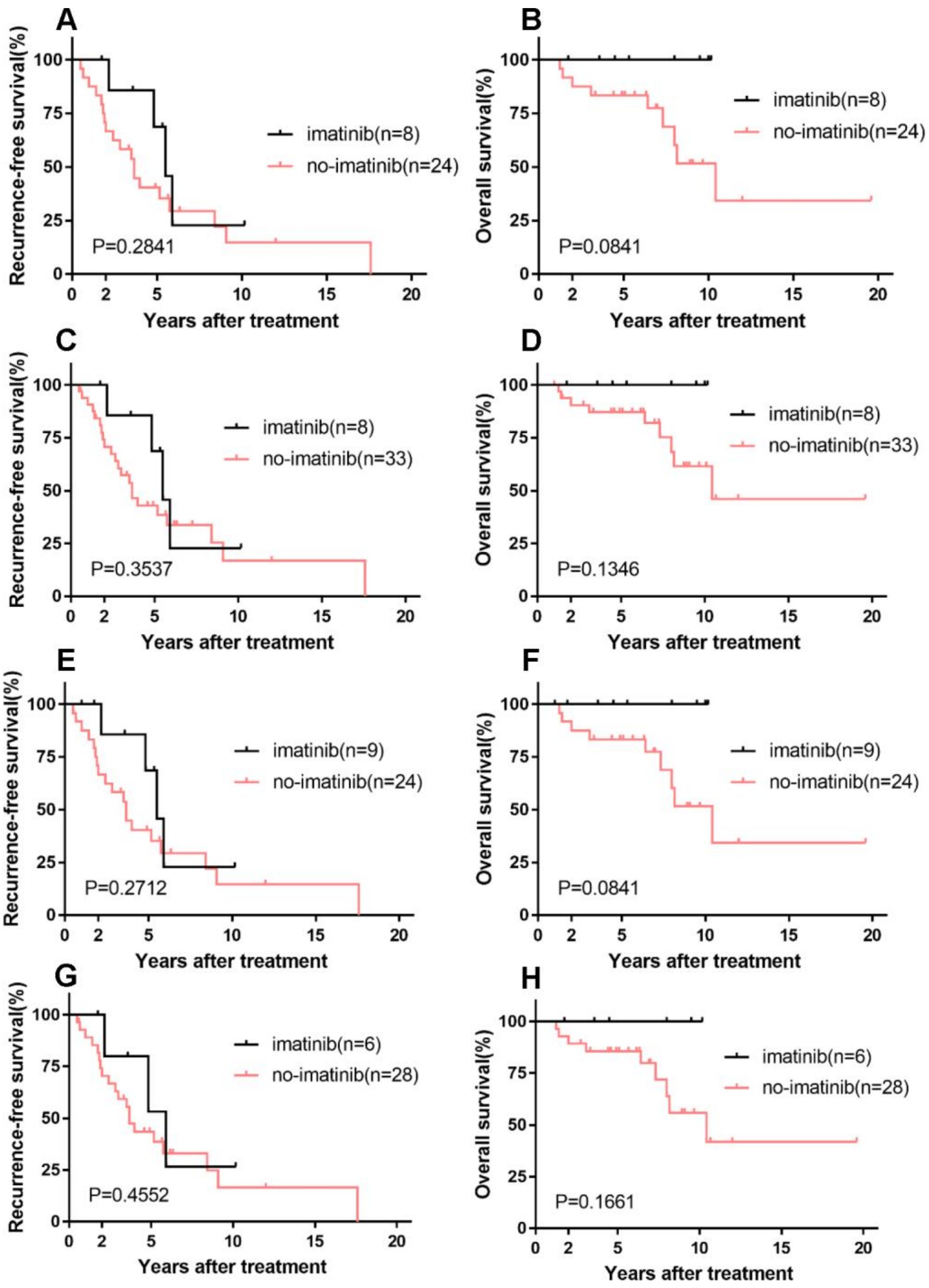


**Supplementary Figure 2. Kaplan-Meier survival plot of OS and RFS based on imatinib in higher risk.** FD-ZS II (**A**, **B**), FD-Hou II (**C**, **D**), WHO III (**E**, **F**), NIH IV (**G**, **H**).
