# Supplementary material for: Comparison of prognostic prediction models for rectal gastrointestinal stromal tumor
Source: Aging (Albany NY). 2020 Jun 20;12(12):11416–30. doi: 10.18632/aging.103204 (PMC7343501; doi:10.18632/aging.103204)
Supplement: Supplementary Table 3 [file aging-12-103204-s005..pdf]

Supplementary Table 3. Cox regression (RFS) based on adjuvant and neoadjuvant.

Characteristic	Variable	Recurrence-free survival	
		Univariate Cox regression	
		OR(95% CI)	P value[a]
Sex	Male	reference	0.330
	Female	0.473(0.105-2.134)	
Procedure of treatment	LE	reference	0.223
	APR/TPE	0.000(0.000-.)	
	Imatinib	1.004(0.111-9.066)	
	Neoadjuvant	5.020(0.507-49.700)	
Center	Out-zhongshan	reference	0.440
	Zhongshan	0.405(0.050-3.302)	
IM after the first surgery	No	reference	0.312
	Yes	2.959(0.361-24.281)	
Cell type	Mixed	reference	0.773
	Spindle	21.593(0.000-.)	
Age	years±SD	1.001(0.949-1.057)	0.962
Distance to AV	cm±SD	0.832(0.485-1.426)	0.503
Time of IM	months±SD	0.965(0.918-1.015)	0.163
Tumor size at diagnosis	cm±SD	1.560(0.930-2.616)	0.092
Mitotic counts(50 HPFs)		0.989(0.950-1.030)	0.595
